# Cytotoxic effect of a new endodontic cement and mineral trioxide aggregate on L929 line culture

**Published:** 2008-04-02

**Authors:** Jamileh Ghoddusi, Jalil Tavakkol Afshari, Zakiyeh Donyavi, Azam Brook, Reza Disfani, Mohammad Esmaeelzadeh

**Affiliations:** 1 *Department of Endodontics, Dental Research Center, Dental School, Mashad University of Medical Sciences, Mashad, Iran and Member of Iranian Center for Endodontic Research*; 2 *Department of Bou Ali Research Institute, Mashad University of Medical Sciences, Mashad, Iran*; 3 *Department of Endodontics, Dental School, Mashad University of Medical Sciences, Mashad, Iran*; 4 *Technician of Bou Ali Research Institute, Mashad university of Medical Sciences, Mashad, Iran*; 5 *Department of Pediatric Dentistry, Dental School, Shahid Beheshti University MC, Tehran, Iran*

**Keywords:** Cytotoxicity test, Fibroblast, New material, Mineral trioxide aggregate

## Abstract

**Introduction:** The aim of this study was to compare the cytotoxicity of Mineral Trioxide Aggregate (MTA) and a New Endodontic Cement (NEC) on L929 mouse fibroblasts.

**Materials and Methods:** Different dilutions (Neat, 1/2, 1/10, 1/100) of fresh and set materials placed adjacent flasks of L929 in DMEM medium. Cellular viability was assessed using MTT assay in three time intervals (24, 48, and 72 h after mixing). Differences in mean cell viability values between materials were assessed by using the One-way ANOVA and Bonferoni post-test. Optical microscopic analysis of morphology of the untreated control and the cement-treated cell cultures were carried out in all experimental periods.

**Results:** It was indicated that there was not a significant difference in cytotoxicity among the materials of test and between them and the control group. However, there was a statistically significant difference between different time intervals within each group (P< 0.05) and between different concentration of test materials (P<0.05). In all samples, set materials showed better viability than fresh ones.

**Conclusion:** According to results of this study, NEC and MTA have similar cytotoxic effect on L929 cell culture.

## Introduction

Properties of a good root-end filling material include the ability to adhere and seal the root canal system. The material also should be easy to manipulate, radiopaque, dimensionally stable, non-absorbable, biocompatible with the periradicular tissue, and nontoxic (1).

MTA is an endodontic material that was developed at Loma Linda University in 1993 (2). This material was first used as a root-end filling, but it has also been used as a viable alternative for various clinical applications, such as capping of pulp tissue, root end closure and for repairing furcal perforations (3). Underlying these applications are the properties of MTA that include biocompatibility, good sealing ability and capability of promoting dental pulp and periradicular tissues regeneration (2). Perez *et al.* reported that MTA might be an ideal material because it consistently induced the regeneration of periodontal ligament tissues, the apposition of a cementum like material and formation of bone (4). MTA has been reported to be biocompatible in many *in vivo* and *in vitro* studies. Koh *et al. *reported that MTA offered a biologically active substrate for bone and cells stimulating interleukin production (5). Mitchel *et al.* reported that MTA was biocompatible and suitable for clinical trials (6). Zhu *et al.* reported that osteoblasts have a favorable response to MTA (7). Although MTA has superior biocompatibility in comparison with other materials, it has delayed setting time (8), poor handling characteristics (9), and is an expensive material.

Recently, a new endodontic cement (NEC) consisting of different calcium compounds was developed by Asgary (10). Clinical uses of this cement are similar to MTA. It has good handling characteristics and forms an effective seal when used as root-end filling material (11). NEC is also able to produce hydroxyapatite (12). The results of an in vivo study showed that as pulp capping materials, MTA and NEC showed similar favorable results. These results were better than calcium hydroxide (13).

So, the purpose of this study was to compare the cytotoxicity of MTA and NEC on L929 mouse fibroblasts 

## Materials and Methods

Test materials used were ProRoot MTA (Dentsply, Tulsa Dental, OK, USA) and a Novel Endodontic Cement. Samples of the materials were prepared under aseptic conditions according to the manufacture and inventor’s direction. The samples were divided into two groups. The first group included all materials in a freshly mixed state, whereas in the second group materials were allowed to set for 24 h at 37ºC at 100% relative humidity.

Extracts of the materials were made as follows: 5 of complete Dulbecco’s Modified Eagles Medium (DMEM) was added to 1gr of test material in every one state (fresh and set), and the tubes were incubated at 37ºC at 100% relative humidity for 24h. The medium was then drawn off and sterilefiltered at 0.22 µm. To observe a dose-response relationship, the extracts were serially diluted with complete DMEM to achieve a total of four concentra-tions (Neat, 1/2, 1/10, and 1/100 V/V). 5-Flurouracil was dissolved in complete DMEM and tested as positive control, complete DMEM placed into empty 96 well tissue culture plates for 24, 48, 72 h was tested as negative control.

L929 (ATCC CCL I, NCTC clone 929, mouse L Cells) mouse fibroblasts were grown in Dulbecco’s Modified Eagles Medium (Sigma chemical co, Germany), in a bicarbonate buffer lsystem, supplemented with 10% (V/V) fetal bovine serum,50 µg/streptomycin and 100 units/ mL Penicillin.

L929 cells were collected by washing with serum free DMEM before treatment with 5 trypsin (0.1%), 1 EDTA (0.1%) solution in phosphate buffered saline for 7-10 min. Cells from the fourth collection were plated in a 96 well plate at a density of 5×103 cells per well and allowed to attach for 24 h to the DMEM plus supplements. The following protocol is adapted from Schweikl and Schmalz and Wataha *et al*. (14-15). Single cell suspensions of L929 fibroblasts were seeded in 96-well flat-bottomed plates, 5×103 cells per well as determined by hemo-cytometer counting, in complete DMEM, and incubated in a humified atmosphere of air and 5% co2 at 34úc for 24h. The culture medium then was replaced with 200 µl aliquots of the test extracts or control media.

Effects of the materials on mitochondrial function were measured by a colorimetric assay as described by Mossman (16). Upon incubation with viable cells, the tetrazolium ring of MTT (Plate yellow) is cleaved by cellular dehydrogenase enzymes to convert the yellow water-soluble tetrazolium salt MTT into dark blue formazan crystals. MTT Solution (0.5 mg/well) was added to each plate and they were incubated to be solubilized with dimethyl-sulphoxide and the absorbance determined at A570nm using an ELISA plate reader (Thermomax Microplate Reader: Molecular devices, Santo Monica, CA, USA). At each experimental time period (24, 48 and 72h), an MTT assay was conducted to measure cell viability. Optical microscopic analysis (Ziess, Germany) of the morphology of the human untreated control and the cement- treated cell cultures were carried out in the five experimental periods (24, 48, 72, 96h, and 6d).

**Figure1:**
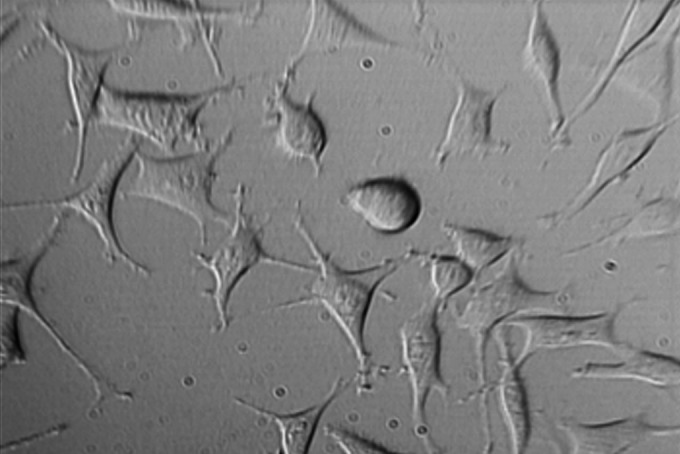
L929 cells exposed to neat concentration of fresh MTA after 24 h.Mag×200

**Figure2:**
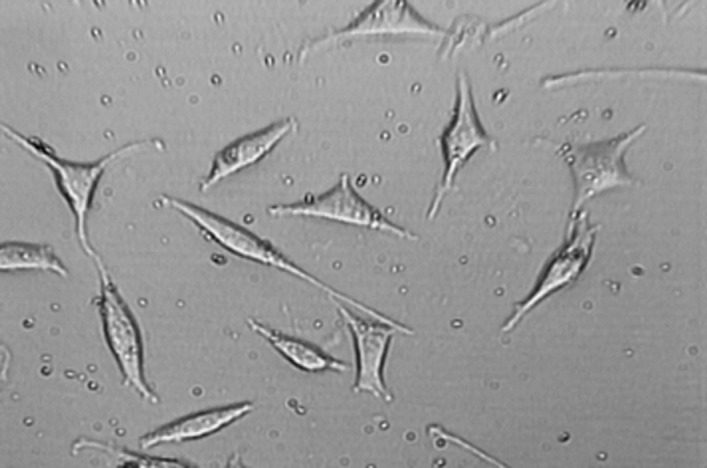
L929 cells exposed to neat concentration of fresh NEC after 24 h.Mag×200

**Figure3:**
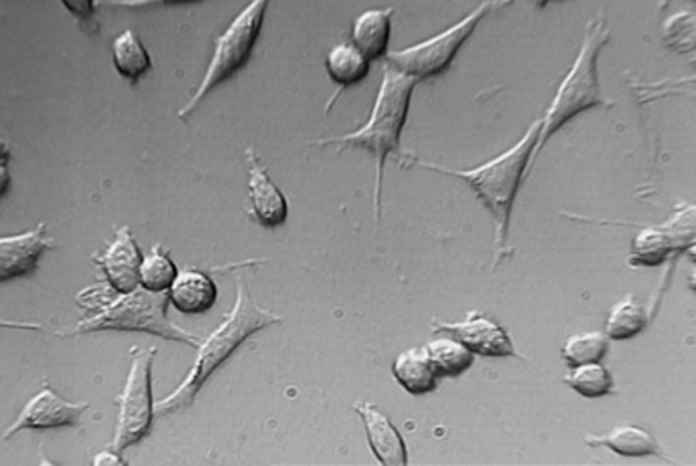
L929 cells exposed to Neat concentration of set MTA after 24h. Mag×200

**Figure4:**
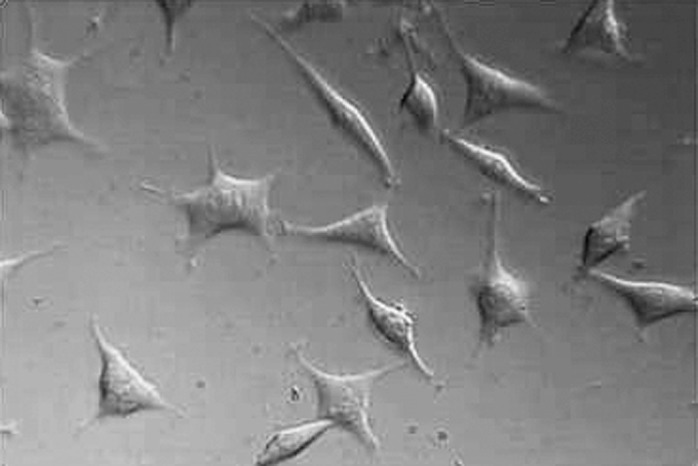
L929 cells exposed to Neat concentration of set NEC after 24h. Mag×200

**Figure5:**
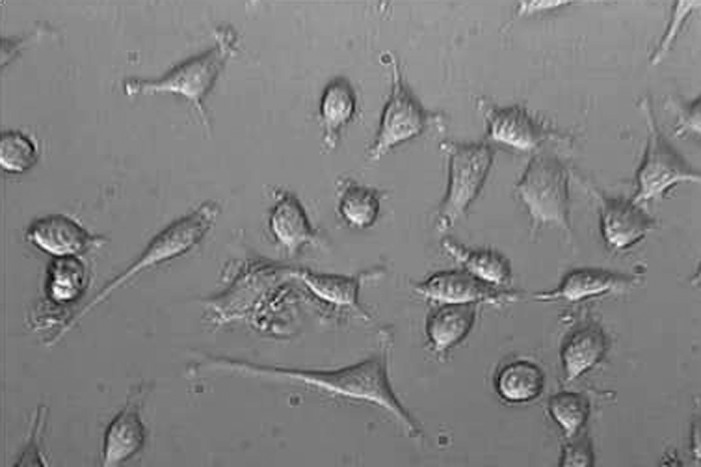
L929 cells exposed to 5-FU in concentration of 16µg/ after 24h. Mag×200

The mean absorbances of the three wells containing the same extract and their standard deviation were calculated. Original optical density values of test cultures were expressed as percentage of optical density obtained from the control medium. The absorption value obtained with the control was considered as indicating 100% viability cytotoxicity. More than 90% cell viability was considered as non- cytotoxic, 60-90% as slightly cytotoxic, 30-59% as moderately cytotoxic and < 30% cell viability was also considered as strongly cytotoxic (16). All assays repeated three times to guarantee reproducibility. One-way analysis of variance and the Bonferoni post-test statistically analyzed the significance of the difference between the control andexperimental groups.

A P<0.05 was considered statically significant.

## Results

Findings of this study revealed that MTA and NEC do not induce cytotoxicity on L929 in both techniques including optical microscopy (Figures 1, 2, 3, 4 and5) and MTT assay. A confluent cell culture was observed in the negative control group maintained for the whole time of the experiment.

**Figure6:**
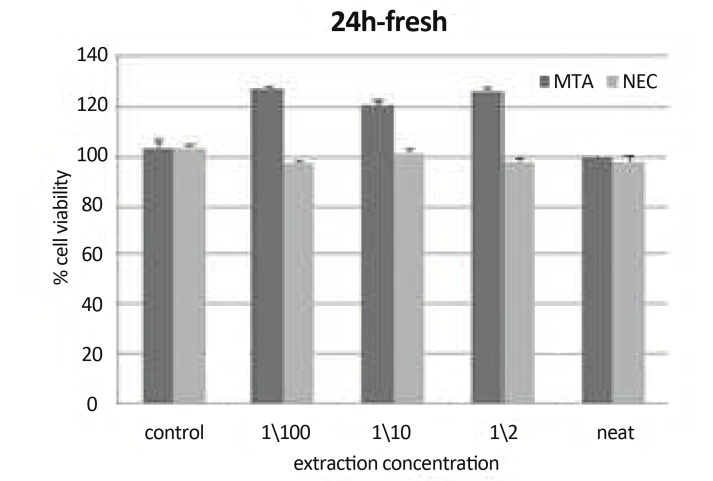
Mean cell viability +SD at different concentration (0 concentration for control) using freshly mixed materials at 24h on L929 by MTT assay.

***In*** ***24h,*** ***Fresh*** ***Materials:*** There were statistically significant differences (P<0.05) between cell viability (CV) of neat MTA and their other concentrations. There was a statistically significant difference (P<0.01) between CV of NEC and MTA in concentration of 1/2 (Figure 6).

**Figure7:**
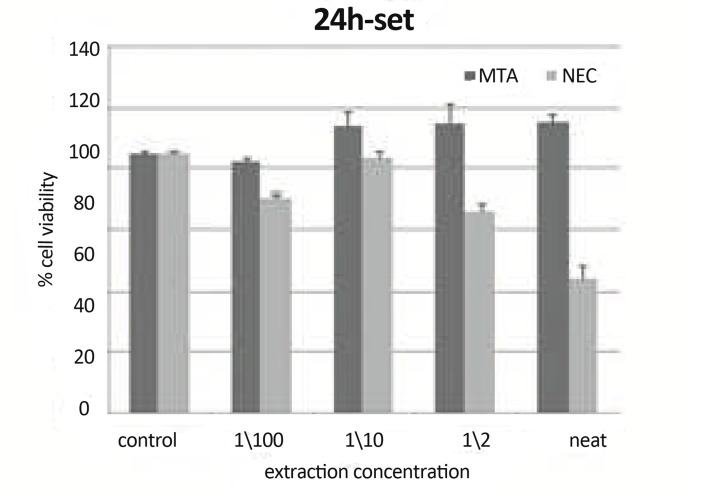
Mean cell viability +SD at different concentration (0 concentration for control) using set materials at 24h on L929 by MTT assay.

***In 24h, Set Materials:*** There were statistically significant differences (P<0.01) between CV of neat NEC and their other concentrations. There was a statistically significant difference between CV of neat NEC and control group (P<0.001). There was a statistically significant difference (P=0.001) between CV of NEC and MTA in concentration of neat (Figure 7).

**Figure8:**
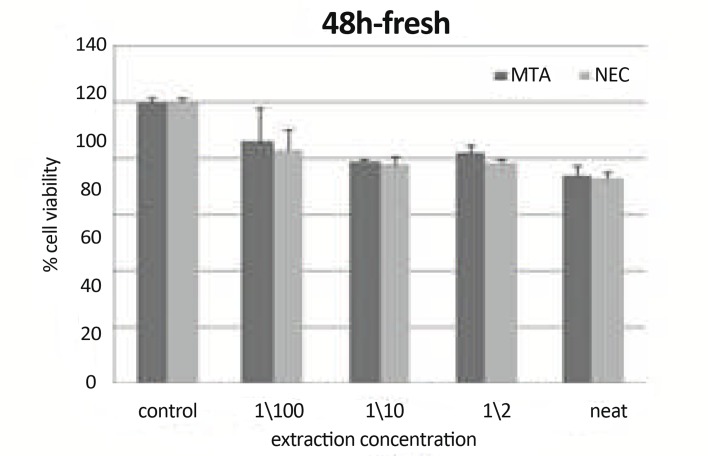
Means cell viability +SD at different concentration (0 concentration for control) using freshly mixed materials at 48h on L929 by MTT assay.

***In 48h, Fresh Materials:*** There were statistically significant differences between CV of Neat, 1/10/, 1/100 concentration of both materials and control group (P<0.05). There were statistically significant differences between CV of Neat of MTA and 1/2, 1/10, 1/100 of it (P<0.05). There was a statistically significant difference between CV of Neat of NEC and 1/ 2, 1/10 of it (P<0.05). There were statistically significant differences between CV of 1/2 of NEC and 1/10, 1/100 of it (P<0.05). There was a statistically significant difference (P<0.01) between CV of NEC and MTA in concentration of 1/2 (P=0.033) (Figure 8).

**Figure9:**
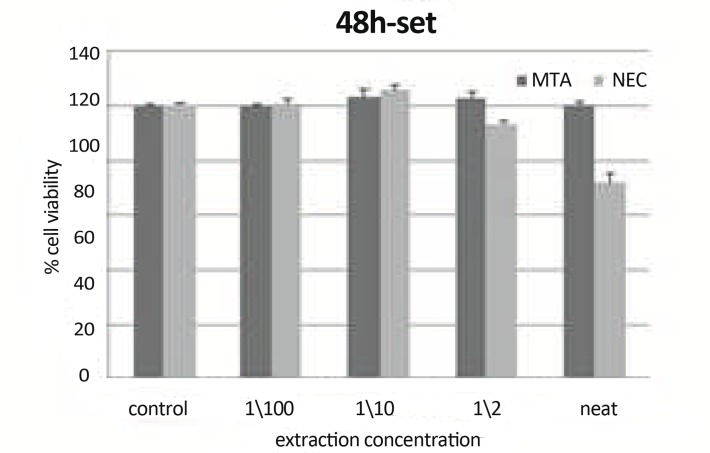
Means cell viability +S at different concentration (0 concentration for control) using set materials at 48h on L929 by MTT assay.

***In 48 h, Set Materials: ***There were statistically significant differences between CV of neat NEC and their other concentrations (P<0.05). There were statistically significant differences between CV of neat of NEC and control group (P=0.001). There was a statistically significant difference between CV of NEC and MTA in concentration of neat (P<0.001). There was a statistically significant difference between CV of NEC and MTA in concentration of 1/2 (P=0.02) (Figure 9).

**Figure10:**
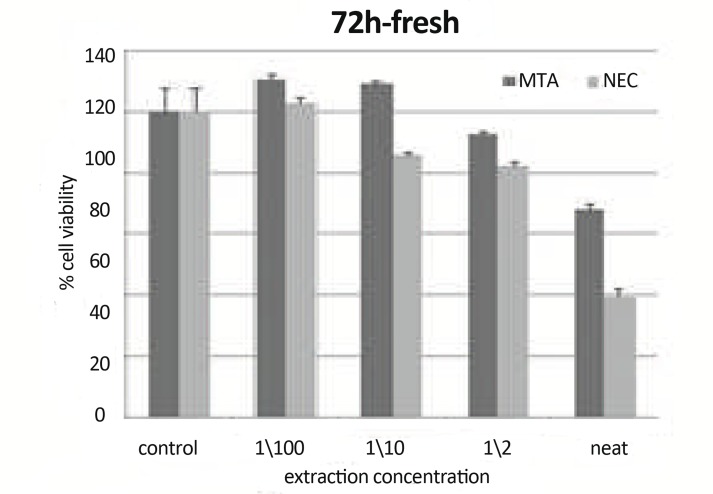
Means cell viability +SD at different concentration (0 concentration for control) using freshly mixed materials at 72h on L929 by MTT assay.

***In 72h, Fresh Materials:*** There were statistically significant differences (P<0.001) between CV of neat MTA and their other concentrations. There were statistically significant differences between CV of neat, 1/10, 1/100 concentration of both materials and control group (P<0.05). There were statistically significant differences (P<0.001) between CV of neat of NEC and their other concentrations. There were statistically significant differences (P<0.001) between CV of materials in concentration of 1/2 and 1/10 and 1/2 and1/100. There was a statistically significant difference between CV of NEC and MTA in concentration of 1/100 (P=0.003) (Figure 10)

**Figure11:**
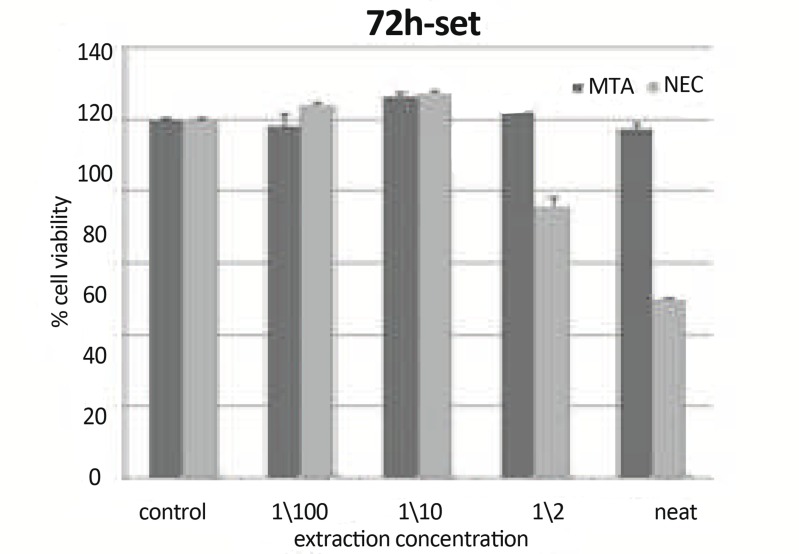
Means cell viability +SD at different concentration (0 concentration for control) using set materials at 72h on L929 by MTT assay.

***In 72h, Set Materials:*** There was a statistically significant difference between CV of NEC and MTA in concentration of neat (P<0.001). There were statistically significant differences between CV of 1/2 and 1/10 and 1/2 and 1/100 concentration of NEC (P=0.02). There were statistically significant differences between CV of NEC in concentration of neat and 1/10 (P=0.002). There were statistically significant differences between CV of NEC in concentration of neat and 1/100 (P=0.003). There was a statistically significant difference between CV of neat NEC and control group (P=0.035) (Figure11).

Comparison of fresh and set state of test materials showed at 24h: There was a statistically significant difference between CV of neat concentration of MTA (P=0.002). There was a statistically significant difference between CV of neat concentration of NEC (P<0.001). There was a statistically significant difference between CV of 1/100 concentration of NEC (P=0.034).

Comparison of fresh and set state of test materials showed at 48h: There was a statistically significant difference between CV of 1/10 concentration of MTA (P=0.017). There was a statistically significant difference between CV of 1/100 concentration of MTA (P=0.001). There was a statistically significant difference between CV of 1/10 concentration of NEC (P=0.0049). There was a statistically significant difference between CV of 1/100 concentration of NEC (P=0.012).

Comparison of fresh and set state of test materials showed at 72h: There was a statistically significant difference between CV of neat concentration of MTA (P<0.001). There was a statistically significant difference between CV of neat concentration of NEC (P=0.029).

## Discussion

In this study cytotoxicity of Pro Root MTA and NEC were evaluated with comparison because these materials have been introduced as a root end filling material and in clinical application are in close contact with live tissue (17). The toxic effects of materials used for endodontic therapy are of particular concern, because damage or irritation could cause degeneration of the periapical tissue and delayed wound healing*.*In* in vivo* tests such as implantation and usage tests have an advantage in that they allow complex interaction between the host and the material to be examined. In* in vitro *tests such as cell culture enable experimental factors and variables to be controlled which often is a significant problem when performing experiments *in-vivo*. These *in vitro* model assays are increasingly being used for initial screening of new dental materials intended forclinical use (17). A variety of test systems are available to determine the cytotoxicity of dental materials in cultured mammalian cell populations. Permeability assays monitor the integrity of cell membranes by the inclusion or exclusion of vital dyes or by the release of radiolabeled chromium. Replication assays indirectly assess the ability of cells to proliferate by measuring the incorporation of nucleotide analogues that have been radiolabeled or are detectable by immunoassay during DNA synthesis. Changes in the cellular cytoskeleton or at the cell surface are observed by morphological studies. Finally, functional assays typically evaluate the cell’s ability to provide the energy necessary for anabolic activities, or the end products of such activities. The assay used in the present study used the tetrazolium salt MTT to measure mitochondrial dehydrogenase activity. It is a plate yellow substrate that produces a dark blue formazan product when cleaved by active mitochondria. Therefore, the reaction only occurs in living metabolically active cells. The decision to use a particular test system should be based on its consonance with the chemical nature of the material being tested. For example, if a material is not likely to cause a change in the permeability of cell membranes, a permeability assay is less apt to determine cytotoxicity in a valid manner. Because MTA is a hydrophilic substance, it is likely to release ionic components. It would be more apt to interfere with intracellular enzyme activities than influence membrane permeabilities (17). Therefore, the MTT assay was chosen for the present study.

After mixing materials, in order to achieve effective dilutions for performing the tests, serial dilution method was used, which is applied for evaluation of dose-response effect in material toxicity studies and was due to Keiser’s method(18). Preparation of Neat concentration (1 gram of test sample with 5 mL of culture media) was due to Ossorio (19).

Eluates (extracts) of the test materials were used in the present investigation. They offer the advantages of being easily sterilized by filtration, and the ability to examine the effect of materials on cells that are both distant to and in contact with them. Sterilization of the test materials for direct contact testing, introduces the possibility of changing the properties of the materials. The use of eluates also simulates the immediate postsurgical root end environment in which toxic elements of the retrofilling material leach into the surrounding fluids in the bony crypt. Eluates can also be made in a series of concentrations to observe a possible dose-response relationship and determine the ideal concentration for the sensitivity of the cells tested (17).

For evaluating toxicity, Pro Root-MTA and NEC were tested in two states of freshly mixed and set. Generally freshly mixed materials as they release materials during chemical setting reactions, have more cytotoxicity. However, when the setting reactions complete, materials whole structure becomes chemically fixed and may have less cytotoxicity. This evaluation was performed according to the method of previous studies (8, 18, 19).

In this study, both qualitative assessment including morphologic evaluation applying optical microscopy and quantitative assessment with cell functional tests were accomplished. Thus, according to quality and quantity assessment in this investigation, it possesses the privilege that what was observed in optical microscope qualitatively was also evaluated quantitatively using MTT assay test. While most of other studies were only based on whether quantitative or qualitative assessment, the histological investigations of Christopher *et al*. (20) on tissue response of dog’s periapical, Torabinejad *et al*. (21) on tissue response of monkey’s periapical incisor, Zhu *et al.* (7) on osteoblast cell response in contact with retrofill compounds, all were qualitative. While, the studies of, Ossorio *et al. *(19) evaluating MTT assay and crystal violet assay, Torabinejad *et al.* (22) based on two techniques of Agar over lay and Radiochromium Release method and Keisser (18) with MTT assay technique, represent quantitative assessment of materials’ cytotoxicity.

NEC contains some constituents such as tricalcium phosphate, calcium sulfate, calcium silicate, calcium hydroxide, calcium oxide and some others, which have been added to NEC for improving histocompatibility and physicochemical properties. Calcium hydroxide can be produced by calcium oxide hydration. Based on the obtained results of this study, calcium hydroxide is not toxic for vital tissue. This finding is in accordance with Das (23) results, but has contradiction with Cox data (24), probability because of the produced calcium hydroxide concentration. No Study has been done on the amount of calcium hydroxide produced from NEC yet. Holland has suggested that MTA forms calcium hydroxide when is in contact with tissue fluid and triggers hard tissue precipitation (25). Findings of this study revealed that MTA and NEC do not induce cytotoxicity on L929 fibroblasts in both techniques including optical microscopy and MTT assay. The obtained result for MTA was in accordance with many other investigations.

## Conclusion

Results of this study encourage us using NEC as an alternative of MTA, but further studies needed to assessing other properties of this material.
